# A Review on the Relationship Between Sound and Movement in Sports and Rehabilitation

**DOI:** 10.3389/fpsyg.2019.00244

**Published:** 2019-02-12

**Authors:** Nina Schaffert, Thenille Braun Janzen, Klaus Mattes, Michael H. Thaut

**Affiliations:** ^1^Department of Movement and Training Science, Institute for Human Movement Science, University of Hamburg, Hamburg, Germany; ^2^Music and Health Science Research Collaboratory, Faculty of Music, University of Toronto, Toronto, ON, Canada

**Keywords:** acoustic feedback, movement sonification, rhythmic auditory stimulation, sports, motor rehabilitation, Parkinson’s disease, stroke

## Abstract

The role of auditory information on perceptual-motor processes has gained increased interest in sports and psychology research in recent years. Numerous neurobiological and behavioral studies have demonstrated the close interaction between auditory and motor areas of the brain, and the importance of auditory information for movement execution, control, and learning. In applied research, artificially produced acoustic information and real-time auditory information have been implemented in sports and rehabilitation to improve motor performance in athletes, healthy individuals, and patients affected by neurological or movement disorders. However, this research is scattered both across time and scientific disciplines. The aim of this paper is to provide an overview about the interaction between movement and sound and review the current literature regarding the effect of natural movement sounds, movement sonification, and rhythmic auditory information in sports and motor rehabilitation. The focus here is threefold: firstly, we provide an overview of empirical studies using natural movement sounds and movement sonification in sports. Secondly, we review recent clinical and applied studies using rhythmic auditory information and sonification in rehabilitation, addressing in particular studies on Parkinson’s disease and stroke. Thirdly, we summarize current evidence regarding the cognitive mechanisms and neural correlates underlying the processing of auditory information during movement execution and its mental representation. The current state of knowledge here reviewed provides evidence of the feasibility and effectiveness of the application of auditory information to improve movement execution, control, and (re)learning in sports and motor rehabilitation. Findings also corroborate the critical role of auditory information in auditory-motor coupling during motor (re)learning and performance, suggesting that this area of clinical and applied research has a large potential that is yet to be fully explored.

## Introduction

In the last decades, research in the fields of sport, neuroscience, and psychology, has sought to better understand the role of sounds on perceptual-motor processes from multiple angles of investigation. In applied research, there has been a great interest in how auditory information affect the production of complex movements and how it may be used in sports training and movement rehabilitation to improve motor performance in athletes, healthy individuals, and patients affected by neurological or movement disorders (e.g., Dubus and Bresin, 2013; Sigrist et al., 2013; Murgia et al., 2015; Pizzera and Hohmann, 2015; Sors et al., 2015; Thaut et al., 2015; Ghai and Ghai, 2018; Ghai et al., 2018a,b). However, this body of research is scattered both across time and scientific disciplines. Therefore, the aim of this paper is to provide an overview about the interaction between movement and sound and review the current literature regarding the effect of acoustic information to improve movement execution, control, and (re)learning in sports and motor rehabilitation.

The first section of the paper (Key topic 1) focuses on sports movements and presents an overview of studies investigating the effect of natural movement sounds and sonification in athlete performance enhancement. Natural movement sounds refer to real-time and naturally occurring acoustic information in the form of auditory reafferences, such as the sound resulting from the contact phase of the feet meeting the ground or the physical impact of limbs or equipment with air/ground/water/ball (Kennel et al., 2015; Pizzera and Hohmann, 2015). Natural auditory signals provide a large amount of information about movements that are readily available to the listener (Gaver, 1993a,b) and may be used in sport training to inform or enhance task-intrinsic feedback (Dubus and Bresin, 2013; Sigrist et al., 2013; Sors et al., 2015). Another line of research is dedicated to the development of perceptual strategies based on auditory information to assist movement execution and control through sonification. Sonification involves the transformation of kinematic and dynamic movement parameters into non-speech artificially produced sounds in order to improve motor perception and performance (Effenberg, 2005).

The second section (Key topic 2) addresses the use of sounds in motor rehabilitation. Firstly, we focus on rehabilitation methods that administer auditory rhythmic cues to improve motor function in Parkinson’s disease (PD) and post-stroke, such as Rhythmic Auditory Stimulation (RAS) (Thaut and Hoemberg, 2014; Murgia et al., 2015). Secondly, we consider studies investigating the application of movement sonification (i.e., real-time artificially produced sounds or musical sonification) to assist in the rehabilitation of motor functions in PD and stroke. Note that musical sonification differs methodologically from music-supported therapy, where the former is a relatively novel approach that uses measuring systems (e.g., inertial sensors) to map different movement parameters using musical components, and the latter involves repetitive exercises using musical instruments to retrain motor functions, thus not providing continuous real-time movement feedback (see for review, De Dreu et al., 2012; Zhang et al., 2016; Sihvonen et al., 2017). Studies addressing background music or applying music as auditory feedback are beyond the scope of this review.

In the third section (Key topic 3), we provide an overview of current evidence regarding the neural mechanisms involved in auditory-motor coupling. Particularly, we describe brain regions involved in auditory-motor coupling and address the role of mechanisms such as auditory-motor entrainment, auditory mirror neurons, and sensorimotor integration. By organizing and providing a critical appraisal of the current research, we attempt to develop a framework for future applied and clinical research on the effects of auditory information for motor control and (re)learning.

## Methods

### Search Strategy

The systematic searches included numerous electronic literature databases (e.g., MEDLINE, EMBASE) and trial registers, as well as hand-searching of major journals, abstract books, conference proceedings and reference lists of retrieved publications. Also, potentially relevant texts known to the reviewers were included.

### Study Selection

**FIGURE 1:**
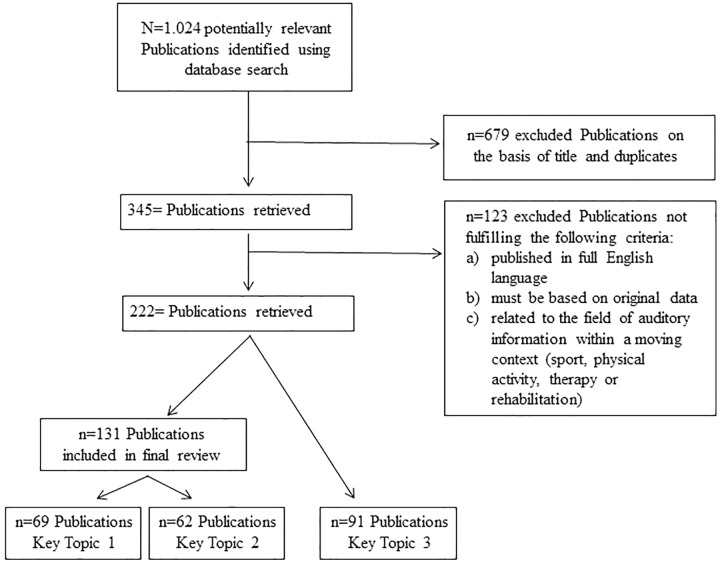
Overview of the search and screening process for the relevant literature.

The search and screening process for relevant literature is shown in **Figure 1**. The titles of all retrieved publications were checked, duplicates were removed, and those publications related to other fields of research were excluded. The initial screening resulted in 345 remaining publications, which were further screened for eligibility based on the following criteria: (a) the work must be published in full in English language, (b) must be based on original data, and (c) must be related to the field of auditory information within the context of sport or sport-related activities, and rehabilitation. Publication abstracts and full texts were used to perform a thorough check of these criteria. After this step, 222 publications were identified and included in this paper, of which 131 papers are clinical or applied studies investigating the effect of auditory information in sports and motor rehabilitation.

## Results

### Key Topic 1: Natural Movement Sounds and Movement Sonification in Sports

#### Natural Movement Sounds

The role of natural movement sounds in auditory action-perception coupling has been studied in sports domains and daily physical activity as part of more general research. Among the topics investigated, studies have examined the influence of natural movement sounds on movement execution (Agostini et al., 2004; Kennel et al., 2015), sense of agency (self vs. other) (Murgia et al., 2012a; Kennel et al., 2014a,b), action anticipation (Cesari et al., 2014; Allerdissen et al., 2017; Camponogara et al., 2017; Sors et al., 2017, 2018a,b; Cañal-Bruland et al., 2018), and motor learning (Pizzera et al., 2017).

Natural movement sounds carry rich auditory information that has direct physical correspondence to their referent event(s), providing crucial information that may be used to inform or enhance task-intrinsic feedback (Dubus and Bresin, 2013; Sigrist et al., 2013; Sors et al., 2015). One of the direct effects of the presence of natural movement sounds is improving athletes’ movement execution, as shown in a study investigating hammer throwing (Agostini et al., 2004). The role of auditory information on movement execution has also been investigated by manipulating the amount or the temporal features of feedback provided to athletes. It has been shown, for instance, that deprivation of auditory feedback hindered the performance of experienced tennis players by decreasing receiving service precision (Takeuchi, 1993). Kennel et al. (2015) examined whether the sounds of the steps during running would influence hurdling performance and found that temporally delayed auditory feedback decreased athletes’ performance by slowing down the time to complete the track and affecting the motion sequence during the first trials where the manipulation was presented. However, there were no differences in movement execution when comparing normal real-time auditory feedback condition and white noise.

Natural movement sounds also provide fundamental information about agency and facilitate the discrimination of one’s own from another person’s movement. The role of specific sound features on the sense of agency has been recently investigated in sports such as golf (Murgia et al., 2012a) and hurdling (Kennel et al., 2014a,b). Murgia et al. (2012a) found that expert golfers could identify the recorded sounds of their own golf swings from those of other athletes based on the temporal features of the movement sound, such as the overall action duration (i.e., how long the swing movement lasted from beginning to end) and the rhythmic patterns of the backswing and downswing movement. Kennel et al. (2014a,b) also found that athletes could distinguish between their own hurdling movements from those of others’ on the basis of the auditory information, using a variety of sound characteristics (e.g., hurdling step structure, amplitude of the sounds) to build a holistic representation of their own and others’ movements.

Research has also shown that athletes are able to extract relevant information from the sounds generated by their own or others’ movements to predict and anticipate actions based on changes in the environment or the opponents’ behaviors. It has been demonstrated, for example, that expert basketball players can detect the movement intentions of an opponent and prediction their running direction based on the sounds generated by the opponent’s movements (Camponogara et al., 2017). Cesari et al. (2014) found that the ability to precisely anticipate and reproduce a skateboarding jump based only on movement sounds was superior for experienced athletes than for non-experts. Specifically, only experts were able to modify their underfoot force and apply muscle synergies that were essentially similar to those used during a real jump on a skateboard only by hearing the movement sounds. Similarly, studies have also demonstrated that auditory information generated by movements may be used to predict a attack movement in fencing (Allerdissen et al., 2017), the shot power in soccer (Sors et al., 2017, 2018a), and the length of volleyball serves (Sors et al., 2018b). These behavioral data collectively indicate that the auditory-motor coupling generated during extensive training significantly interacts with athletes’ internal motor simulation as experienced athletes are not only able to extract highly specific information from action-related sounds but also use this information to anticipate another person’s movements based on action prediction mechanisms.

The short- and long-term effects of acoustic reafference to improve movement control and learning of complex movements has been recently investigated. Pizzera et al. (2017) tested a training protocol where natural step sounds produced during hurdling were recorded and presented before each trial with modulated tempo in relation to baseline: faster tempo, slower tempo, or normal tempo. Results showed that the presentation of the auditory information increased overall performance for all groups at short-term, enhancing running time and movement technique. When considering the long-term effects, findings suggested that only the groups that received acoustic information with modulated tempo (faster or slower) further increased performance at a 10-week retention test, whereas the performance of the group who trained with normal auditory feedback declined. These results indicate that, while acoustic information during training have immediate effects on athletes’ performance, repeated training with modified temporal acoustic information may be more effective and contribute to the development of a richer internal representation of the movement.

#### Movement Sonification

Sonification, as the transfer of movement data into non-speech audio signals, refers to the mapping of physiological and physical data onto psychoacoustic parameters (i.e., loudness, pitch, timbre, harmony and rhythm) in order to provide on- and/or offline access to biomechanical information otherwise not available (for an overview see Effenberg et al., 2011, 2016; Dubus and Bresin, 2013; Sigrist et al., 2013; Kos et al., 2015; Pizzera and Hohmann, 2015). Movement sonification thus aims to assist movement control, execution, and planning by improving self-awareness of physiological processes underlying movement execution and optimizing movement regulation and control (Effenberg, 2005).

The potential use of real-time movement sonification has motivated researchers to investigate the effectiveness of sonification as additional real-time acoustic information in sport training to enhance athletic performance in a wide range of sports (see **Supplementary Table 1**), including: running (Eriksson and Bresin, 2010; Bolíbar and Bresin, 2012; Boyd and Godbout, 2012; Sanderson and Hunt, 2016), aerobics (Hermann and Zehe, 2011), rowing (Dubus and Bresin, 2010; Schaffert and Mattes, 2011; Wolf et al., 2011; Cesarini et al., 2014b), swimming (Hermann et al., 2012; Cesarini et al., 2014a), sailing (Tarnas and Schaffert, 2017), cycling (Sigrist et al., 2016; Schaffert et al., 2017), speed skating (Godbout and Boyd, 2010; Stienstra et al., 2011; Boyd et al., 2012; Godbout et al., 2014), skiing (Kirby, 2009; Hasegawa et al., 2012), golf (Kleiman-Weiner and Berger, 2006; Nylander et al., 2014), juggling (Bovermann et al., 2007), German wheel (Hummel et al., 2010), squat jumps (Newbold et al., 2017), motorsport (Powell and Lumsden, 2015), recreational sports (Barrass et al., 2010), postural control (Avissar et al., 2013), slackline (Anlauff et al., 2013), handball (Höner et al., 2004), basketball (Ramezanzade et al., 2014), elastic trampoline (Pugliese and Takala, 2015), and manual wheelchair training and operation (Almqvist Gref et al., 2016).

Investigations examining the use of sonification in elite or high-performance sports have demonstrated that the presentation of artificially generated sounds optimize movement control and execution (e.g., stability, velocity, pattern and force symmetry) in sports such as swimming (Chollet et al., 1988, 1992), rowing (Schaffert et al., 2010, 2011; Schaffert and Mattes, 2011, 2015b, 2016), and cycling (Sigrist et al., 2016; Schaffert et al., 2017). For instance, Chollet et al. (1988, 1992) examined the effects of the presentation of concurrent auditory signals of hydrodynamic pressure exerted by the athlete’s hand during the propulsive action in crawl swimming. Movement data were transformed into auditory signals of equal amplitude and mapped on to pitch so that higher pressure was displayed as a higher pitch. The study results indicated that real-time sonification allowed swimmers to maintain stroke velocity improving movement stability and control (Chollet et al., 1988, 1992). Schaffert and colleagues investigated the influence of acoustic feedback in elite rowing (Schaffert et al., 2010, 2011; Schaffert and Mattes, 2011, 2015b, 2016) and elite para-rowing (Schaffert and Mattes, 2015a). For that, we measured the propulsive boat acceleration trace and converted this information into pitch changes so that athletes perceived an increase in pitch the more the boat accelerated. These studies repeatedly found that movement sonification led to faster boat speeds, increased distances traveled per stroke, and improved crew synchronization compared to training without additional auditory information (Schaffert et al., 2010, 2011; Schaffert and Mattes, 2011, 2015a,b, 2016). In cycling, Schaffert et al. (2017) demonstrated that the continuous real-time auditory information allowed cyclists to perceive fluctuations in forces applied on the pedals and consequently adapt muscle activation to maintain a consistent movement execution pattern and symmetry.

Real-time auditory signals may also enhance athletes’ self-awareness during movement execution by providing auditory feedback otherwise not available. This has been shown in studies evaluating the effect of sonification on exerted muscle power in resistance training and weightlifting (Murgia et al., 2012b; Yang and Hunt, 2013, 2015), precision rifle shooting (Konttinen et al., 2004) and inter-limb coordination in gymnastics (Baudry et al., 2006). Yang and Hunt (2013, 2015) examined the potential of real-time sonification to improve the quality of resistance training. Muscular activity (biceps curl) was measured with electromyographic sensors and sonified in relation to the biceps contractions and extensions so that the more effort was exerted the brighter the tone of the sound. The results showed that the auditory information provided concomitant with the movement helped athletes to maintain the pacing of their movement and improve exercise metrics with greater average repetition range and total effort. Murgia et al. (2012b) also showed that high-intensity sounds presented during the bench-press phase of weightlifting facilitated exerted mean muscle power compared with no sonification. Konttinen et al. (2004) investigated the effects of augmented auditory feedback on precision rifle shooting. The auditory signal informed shooters about rifle alignment by mapping the distance between their aiming point and target center. The study results showed improved shooting performance in the post- and retention tests (after 10 and 40 days) compared to a control group, suggesting that the auditory feedback enhanced shooters’ ability to detect errors in body alignment and modify their movements to improve rifle stability and shooting precision. The presentation of auditory information not usually available to athletes’ also improved inter-limb coordination in gymnastics. Baudry et al. (2006) examined the effects of auditory concurrent feedback on body segmental alignment and inter-limb coordination on experienced male gymnasts during the performance of a circle on a pommel horse. A two-part device (with one piece placed on the upper back and the other – a spring – placed on the knee’s backside, both linked with a cable) informed gymnasts about the bent position of the body with an auditory signal. Positive effects on body segmental alignment were found after 2 weeks of training, with gymnasts in the experimental group improving their percentage of maximum body segmental alignment whereas no gains in body alignment were observed for the control group.

Sonification has also been applied during sports training to inform athletes about performance error/deviation in real-time. Collectively, studies in sports training such as handball (Höner et al., 2004), sailing (Tarnas and Schaffert, 2017), speed skating (Godbout and Boyd, 2010; Godbout et al., 2014), and basketball (Ramezanzade et al., 2014) indicate that the availability of real-time auditory feedback enhances online error-correction mechanisms during movement execution and facilitate the learning of a new motor skill. In speed skating, for instance, Godbout and Boyd (2010) provided corrective sonic feedback to an elite athlete with difficulties to perform the cross-over stride movement. The skating stride was matched to a model skater and the differences were sonified. Based on this sonification model it was possible to provide warning cues, timing, and body position information in real-time, allowing the athlete to make corrections and adjustments during movement execution. Sonification modeling was also tested to improve jump shot in basketball with 20 novice participants (Ramezanzade et al., 2014). For that, one group received visual information from a professional player (model) as well as additional auditory information derived from the angular speed of the elbow joint of the player, whereas the second group only received visual information from the player. The findings indicated that the group who received audiovisual information outperformed the group that received only visual information in both the acquisition and retention tests, suggesting that auditory information may facilitate the acquisition and retention of a new motor skill.

Research indeed suggests that real-time auditory feedback supports the learning and retention of new motor skills. Studies collectively indicate that the acquisition of a new skill or movement technique (e.g., swimming stroke technique, precision shooting, inter-limb coordination in gymnastics, rowing technique, and basketball jump shots) is facilitated when auditory information is provided during the acquisition of a new motor skill (e.g., Chollet et al., 1992; Konttinen et al., 2004; Baudry et al., 2006; Ramezanzade et al., 2014; Schaffert and Mattes, 2014). Moreover, with ongoing training, the sonification of the movement is integrated into an internal representation of that skill, thus enhancing the efficacy of motor learning (Effenberg et al., 2016).

### Key Topic 2: Rhythmic Auditory Stimulation and Movement Sonification in Rehabilitation

#### Rhythmic Auditory Stimulation (RAS)

Rehabilitation programs use rhythmic auditory cues as a means to enhance auditory-motor synchronization and promote sustained functional changes to movement (e.g., Thaut, 2005; Thaut and Hoemberg, 2014; Murgia et al., 2015). In particular, rhythm-based techniques use rhythmic patterns to prime the motor system by providing continuous time references that generate expectations for when auditory events will occur or when a movement needs to be performed. The foreknowledge of the duration of the cues allows movement anticipation and motor preparation, hence increasing the quality and precision of the motor responses (Thaut et al., 2015). Specifically, RAS is a rehabilitation technique that involves the utilization of rhythmic cues (metronome or rhythmically accentuated music with embedded metronome clicks) to facilitate rehabilitation of intrinsically rhythmical movements (Thaut et al., 1999; Thaut and Hoemberg, 2014). RAS can be used as an immediate entrainment stimulus providing rhythmic cues during movement or as a facilitating stimulus for training to achieve more functional movement patterns. This technique typically uses simple metronome beats matched to the patient’s baseline gait, but walking cadence can also be facilitated by using metronome beats embedded in musical patterns that are 5–10% faster than baseline (Thaut et al., 1996). Alternative versions of RAS include metronome sounds embedded in expert-selected (McIntosh et al., 1997) or patient-selected music (Thaut et al., 1996). In these studies, it was proposed that the musical texture would provide additional timing information compared with metronome alone, thus facilitating detection, anticipation, and synchronization to the beat (Thaut et al., 1997). A modification of RAS can also be found in the literature as Rhythmic Auditory Cueing (RAC), which is defined as the application of repetitive isochronous beats. Although the terminology may differ in different disciplines, the basic underlying principle of these techniques is the same.

There is robust evidence of the effectiveness of RAS to improve movement in PD patients (reviewed in Rubinstein et al., 2002; Lim et al., 2005; Rochester et al., 2010; Thaut and Abiru, 2010; Spaulding et al., 2013; Wittwer et al., 2013; Rocha et al., 2014; Schaefer, 2014; Murgia et al., 2015; Thaut et al., 2015; Ghai et al., 2018b), stroke (for review, Thaut and Abiru, 2010; Yoo and Kim, 2016), traumatic brain injury (e.g., Hurt et al., 1998), multiple sclerosis (e.g., Conklyn et al., 2010; Shahraki et al., 2017; reviewed in Ghai and Ghai, 2018), and cerebral palsy (e.g., Kwak, 2007; Kim et al., 2011; Baram and Lenger, 2012; Kwak and Kim, 2013; Ghai et al., 2018a for an overview). As the scope of this paper does not allow for a thorough description of all relevant clinical literature using RAS on motor rehabilitation, here we provide a brief overview of representative clinical studies using RAS in PD and post-stroke.

##### Parkinson’s disease

Gait disturbances such as shuffling, freezing of gait, instability (asymmetry and variability between steps) and general difficulties in walking movements and posture are among the most apparent symptoms of PD (Bloem et al., 2004; Rodger and Craig, 2016). Typically, PD patients with impaired gait have difficulty in regulating stride length (Morris et al., 1996) and tend to walk with reduced velocity and increased cadence or step rate (Knutsson, 1972). One probable origin of gait impairment in PD is deficient internal motor timing mechanisms due to basal ganglia dysfunction. Studies have also suggested that the irregular timing of walking pace may be associated with disturbances of coordinated rhythmic locomotion (Ebersbach et al., 1999; Thaut et al., 2001; Skodda et al., 2010) and sensorimotor synchronization (Bieńkiewicz and Craig, 2015, 2016).

Thaut et al. (1996) first described the effect of rhythmic entrainment on gait patterns in PD by demonstrating that patients who underwent 30 min of daily home-based gait training with RAS significantly improved their gait velocity, stride length, and step cadence after 3 weeks of intervention in relation to controls. These findings were later confirmed by several studies (e.g., McIntosh et al., 1997; Freedland et al., 2002; Del Olmo et al., 2006; Nieuwboer et al., 2007; Arias and Cudeiro, 2008, 2010; Hove et al., 2012; Song et al., 2015; Pau et al., 2016). Studies have also found that RAS training can have positive carry-over effects on movement from a few minutes to up to 4 weeks (McIntosh et al., 1997, McIntosh et al., 1998; Nieuwboer et al., 2009). Other beneficial outcomes include increase in the symmetry of muscle activation in upper and lower limbs (Malcolm et al., 2009; Bailey et al., 2018), and reduction of timing variability (Miller et al., 1996), resulting in more stable walking (Thaut et al., 1999; Hausdorff et al., 2007; Hove et al., 2012). A recent study also found positive effects of RAS for the facilitation of gait relearning (Uchitomi et al., 2013). Additionally, there are indications that RAS is superior in maintaining gait performance during dual-tasks due to low cognitive attentional load (Baker et al., 2008). This robust body of literature has been recently summarized and analyzed in systematic and meta-analysis studies, which concluded that rhythmic auditory information is generally an effective therapeutic tool for treating gait disturbances in PD (see Spaulding et al., 2013; Schaefer, 2014; Ghai et al., 2018b).

Although the application of rhythmic auditory information in gait training is well-established, the use of rhythm-based interventions to improve PD symptoms such as freezing of gait and risk of falls is still under investigation. In relation to freezing of gait, Willems et al. (2006) found no beneficial effects of RAS on freezing of gait in patients with less severe symptoms, but Delval et al. (2014) and Plotnik et al. (2014) reported positive effects of RAS on gait initiation and freezing of gait in PD patients. A recent review (Ginis et al., 2017) also concluded that cue-augmented training can reduce the severity of freezing in PD patients, but limitations in long-term consolidation and transfer of the effects to untrained tasks need to be considered in this population. RAS has been also recently applied to reduce falls or risk of falls in healthy elderly (Hurt-Thaut, 2014) and PD patients (Thaut et al., 2018). These studies collectively found that RAS training significantly reduced the number of falls in healthy individuals and PD patients by modifying key kinematics in gait control, thus suggesting that RAS may be beneficial to address the risk of falls.

A recent line of research has focused on whether specific parameters of the acoustic cues can influence the results of rhythm-based interventions for PD by comparing, for instance, differences between music and isochronous sounds (i.e., metronome) with interactive cueing systems that adapt to the patient’s gait (see review in Ashoori et al., 2015; Hove and Keller, 2015). For instance, Murgia et al. (2018) compared whether the nature of the stimulus presented would influence the effectiveness of RAS by providing ecological footstep sounds as auditory information. For that, one group of PD patients completed 5 weeks of supervised rehabilitation training that included walking while listening to ecological footsteps sounds, whereas the second group of patients walked listening to artificial stimuli (e.g., metronome). The overall conclusion of the study was that biological motion sounds such as footsteps are as effective as the metronome, but exploratory analyses of biomechanical measures suggested that there may be some differences in improvement linked to the type of auditory stimuli. Similarly, Dotov et al. (2017) tested biological variability in auditory stimulus vs. isochronous cues and found superiority of biologically variable auditory cues in fostering natural gait variability in PD patients; however, the authors limited their analysis to only immediate and likely transient effects of cueing. Young et al. (2016) found that action-relevance was a more dominant factor in facilitating improvements in gait parameters than acoustic continuity. Finally, Baram et al. (2016) examined the effectiveness of a device that provided a clicking sound generated in response to every step taken by the patient and found that closed-loop auditory feedback produced better results than open-loop auditory cues (e.g., metronome) in relation to gait speed. It is important to note, however, that the use of closed-loop auditory feedback information stands in contrast to metronome-based approaches in relation to a critical component, that is, the use of external auditory cues as predictable feedforward information transmitted by the steady rhythmic information.

Negative effects of RAS were reported when auditory cues were presented at rates much slower (e.g., 20%) or much higher than the patient’s preferred gait (Del Olmo and Cudeiro, 2005; Nombela et al., 2013). Arias and Cudeiro (2008) and Dalla Bella et al. (2017) also suggested that RAS efficacy may depend on individual characteristics, including severity of disease symptoms and impaired ability to synchronize to the beat. However, there are indications that beat perception may be of lesser importance due to evidence of the primacy of period entrainment over phase/beat entrainment during small tempo perturbations (e.g., Thaut et al., 1998a,b; Roberts et al., 2000; Thaut and Kenyon, 2003).

Overall, research using RAS and rhythmically enhanced-music show consistent evidence of the improvement of motor function in PD. Moreover, recent studies have extended the application of RAS to other non-motor functions (for review, see Thaut and Abiru, 2010). For instance, studies have indicated that RAS training enhances patients’ performance in both motor timing (movement synchronization, tapping) and in perceptual timing tasks (duration discrimination, beat detection in music), supporting the hypothesis that RAS engages brain networks involved in both perceptual and motor timing (Benoit et al., 2014; Dalla Bella et al., 2015).

##### Stroke

Motor impairment is one the most widely recognized consequences of stroke, which include reduced movement coordination, decreased postural control, and decreased upper-limb function (Langhorne et al., 2009). Such significant impairments in locomotive function can lead to limitations in independent mobility, thus strongly affecting patients’ quality of life (Michael et al., 2005).

There is strong evidence that RAS can be effectively applied for timely motor control during gait training for stroke patients (Thaut et al., 1993, 1997, 2007; Hayden et al., 2009; for review see Thaut and Abiru, 2010; Hollands et al., 2012; Thaut and McIntosh, 2014; Nascimento et al., 2015; Yoo and Kim, 2016). Thaut et al. (1993) found that patients who walked with RAS matched to their baseline gait cadence showed decreased stride time variability and more balanced muscular activation pattern between the paretic and non-paretic limbs. Recent studies also indicate significant effects of RAS on standing balance (Suh et al., 2014), and gait coordination and symmetry during normal overground walking (Prassas et al., 1997; Roerdink et al., 2011; Lee et al., 2012; Yang et al., 2016) and treadmill training (Roerdink et al., 2007; Park et al., 2015; Yoon and Kang, 2016; Mainka et al., 2018). Immediate effects of RAS training with tempo changes were also found on gait kinematics (Cha et al., 2014) and in relation to the lesion site (Kobinata et al., 2016). Finally, there is growing support for the use of RAS in gait training during the chronic phase of stroke to improve walking speed and functional mobility (e.g., Shin et al., 2015; Ko et al., 2016; Wright et al., 2016, 2017).

Studies have also reported significant improvements in upper limb function after training with RAS (e.g., Whitall et al., 2000; Thaut et al., 2002a,b; Luft et al., 2004; Jeong and Kim, 2007; Malcolm et al., 2009; Chen et al., 2016). For instance, Malcolm et al. (2009) reported a significant decrease in compensatory reaching movements after a 2-week RAS training program, which consisted of patients moving between at least two targets by touching the digits of their affected hand to the assigned targets in synchrony with the auditory rhythmic stimuli. Another line of interventions has used RAC to prime and facilitate bilateral arm training, also known as BATRAC (for review Wolf et al., 2014; Choo et al., 2015). As an example of BATRAC training, in Whitall et al. (2000) participants pushed and pulled bilaterally two independent bar handles in synchrony or alternation with rhythmic auditory cues. The authors found significant improvement in isometric strength, range of motion, and functional motor performance of the paretic arm after 6 weeks of intervention and also at an 8-week follow-up assessment. Additionally, there are indications that music-supported training using musical instruments can improve motor recovery of arm movements after stroke by inducing auditory-sensorimotor co-representation of movements (e.g., Thaut et al., 2002a,b; Schneider et al., 2007, 2010; Rodríguez-Fornells et al., 2012; Altenmüller et al., 2009; Altenmüller and Schlaug, 2013; Amengual et al., 2013; for a review on music-support training, see Zhang et al., 2016).

#### Movement Sonification

Technology-assisted therapy and rehabilitation seek to help patients in regaining the ability to independently perform daily activities and to facilitate their reintegration into social and domestic life by using advances in smart technologies or robotics (for reviews on robotic-assisted therapy, see Lum et al., 2002; Prange et al., 2006; Kwakkel et al., 2008; Marchal-Crespo and Reinkensmeyer, 2009; Secoli et al., 2011; Pennycott et al., 2012; Rosati et al., 2013).

One of the first applications of sonification in a rehabilitation context was developed by Pauletto and Hunt (2006, 2009). They sonified muscular activity using the temporal patterns in electromyography (EMG) by converting electrical impulses from muscles into auditory information. The goal of this sonification approach was to assist therapists to audibly analyze the complex signals originating from multiple EMG-sensors during physical activity. Several sonification methods and system prototypes have been developed in recent years (e.g., Chiari et al., 2005; Dozza et al., 2005, 2007; Vogt et al., 2010; Tissberger and Wersenyi, 2011; Matsubara et al., 2012; Franco et al., 2013; Torres et al., 2013; Ghai et al., 2018c; see **Supplementary Table 2** for details). A growing body of recent research generally agrees that sonification is a promising feedback tool for patients and therapists, complementing existing analytical components in therapy (such as visual displays) (for an overview, see Huang et al., 2006; Dubus and Bresin, 2013). The following sections present an overview of current investigations in sonification for movement rehabilitation in PD and stroke.

##### Parkinson’s disease

There has been growing interest in the application of sonification systems in neurologic rehabilitation focusing on improving gait in PD patients (e.g., Batavia et al., 2001; Miyake, 2009; Torres et al., 2013; Contreras Lopez et al., 2014; Young et al., 2014; Horsak et al., 2016; Schedel et al., 2016; see **Supplementary Table 2** for details). A sonification system that has received significant attention in recent years is the use of instrumented footwear (Bresin et al., 2010; Fischer et al., 2017; see review in Maculewicz et al., 2016). These systems comprise of interactive shoes with embedded sensors that collect gait information (e.g., cadence, velocity, stride length), which are then used to trigger auditory cueing stimuli to inform both the therapist and the patient about the user’s current state. Recently, Gorgas et al. (2017) tested the effect of an instrumented shoe-insole-device for real-time sonification of gait (SONIGait; see also Horsak et al., 2016). This sonification system mapped individual walking characteristics on to musical notes in order to provide gait spatiotemporal information. Results indicated that a 5-min practice phase with sonification increased gait velocity and cadence, opening the possibility for further testing of this real-time sonification device in large controlled trials. Rodger et al. (2014) tested two sonification systems using synthesized walking sounds to guide and improve gait coordination in PD. The first approach used computer-generated sounds of footsteps on gravel in order to convey ecological information regarding step lengths and duration, whereas the second approach involved real-time sonification of the swing-phase of gait by using motion-capture and audio processing software. Study results suggested that both methods had an effect on step length variability but did not alter step duration variability, suggesting that the presentation of auditory information within the patient’s normal step duration range had an effect only on spatial characteristics of gait rather than temporal parameters.

A recent innovative line of motor learning based interventions have combined action observation and sonification to improve freezing of gait (see Gilat et al., 2018 for review). For instance, Mezzarobba et al. (2018) presented videos showing an actor performing gait-related gestures while simultaneously presenting the sonification generated by the kinematics of each gesture. Patients were then asked to imitate the movements shown. This training protocol was completed twice a week for a total of 8 weeks by a group of 12 patients, whereas the control group practiced the motor gestures by means of visual (stripes on the floor) or auditory cues (metronome). Assessments conducted after the intervention and 3 months after the treatment suggested that the multisensory treatment significantly reduced the number of episodes and duration of freezing facilitating the priming effect generated by action observation, whereas no significant difference was observed for all mobility indices in the control group.

##### Stroke

External real-time auditory feedback has been extensively applied in upper-limb rehabilitation post-stroke (e.g., Maulucci and Eckhouse, 2001; Chen et al., 2006; Wallis et al., 2007; Dailly et al., 2012; Immoos et al., 2013; Bruckner et al., 2014; Fujii et al., 2016; reviewed in Ghai, 2018; see **Supplementary Table 2** for details). For instance, Chen et al. (2006) and Wallis et al. (2007) tested a real-time multimodal sonification system which provided visual and auditory information in order to motivate arm reaching training for stroke patients. Specifically, arm movements triggered musical feedback that provided information about movement smoothness/jerkiness and speed of reach such that the acceleration of the motion during reaching and returning changed the musical intervals and harmonic progressions presented. Test results with three stroke patients reported in Wallis et al. (2007) suggested the feasibility of such sonification systems, opening new avenues for the application of this system in large-scale studies.

Scholz et al. (2015, 2016) investigated the effectiveness of a musical sonification therapy protocol to train gross motor function of upper extremities. For that, patients’ arm movements were sonified in real-time using two inertial sensors placed at the wrist and upper-arm of the affected side. The 3D-movement data were transformed into sounds so that upward movements resulted in an ascending C major scale, vertical movements into changes in brightness/timbre of the sounds, and sagittal movements into changes in loudness. The final goal of the training was to teach patients to play simple melodies by moving their arm in a 3D-sonification space. Patients received an average of 10 days of musical sonification therapy or a sham sonification training that did not include auditory feedback. The study results indicated that patients in the music group improved in measures of motor function relating to the smoothness of reaching but no significant changes were observed in other arm-function measures. Additionally, findings suggested a reduction of joint pain in a subgroup of patients who presented lower pain scores prior to the commencement of the musical sonification therapy.

Schmitz et al. (2014) tested an expanded concept for sonification in upper-limb stroke rehabilitation which included a mobile sonification system that provided 4D information about arm positions and trajectories as captured by inertial sensors. Specifically, hand position was mapped onto four acoustic parameters: arm velocity was mapped onto amplitude; elevation angle onto frequencies between 133.3 and 266.6 Hz; radial arm amplitude changed the impression of sound brightness; and azimuth angle determined the interaural intensity difference. Test results with seven patients indicated the potential application of this sonification system in larger clinical trials (see Schmitz et al., 2018).

Robertson et al. (2009) investigated the effect of sonification on upper limb movements after stroke. Patients performed a reaching task that involved reciprocal pointing to 9 targets while a sensor fixed to the hand processed online kinematic data and modulated the auditory feedback presented during movement. The study reported that the sonification had a positive effect on movement performance such as movement smoothness and trajectory curvature for patients with right hemisphere damage, while it worsened the performance of patients with left hemisphere damage. This result thus suggests that responses to auditory feedback may differ when the side of the lesion after stroke is taken into consideration.

### Key Topic 3: Cognitive Mechanisms and Neural Correlates Underlying Auditory-Motor Coupling

There is robust evidence from multiple lines of inquiry that auditory information has a profound effect on the motor system. Physiological and neuroimaging research has demonstrated that one of the factors underlying this strong interaction is the widely distributed neuroanatomical network connecting the auditory and motor systems at the spinal cord, subcortical and cortical levels (Nayagam et al., 2011; Theunissen and Elie, 2014; Bizley, 2017). For instance, studies investigating reflexive motor responses to sound have described neural pathways formed by descending (efferent) fiber tracts originating in the ventral cochlear nucleus that project bilaterally to sensorimotor tracts in the spinal cord via reticulospinal connections (Rossignol and Melvill Jones, 1976; Huffman and Henson, 1990; Delwaide and Schepens, 1995; Marinovic et al., 2014; Marinovic and Tresilian, 2016). Neuroimaging research has also identified rich neuroanatomical interconnectivity between several distant cortical and subcortical brain areas, including the cerebellum, basal ganglia, thalamus, supplementary motor area (SMA) and pre-SMA, premotor cortex, and the auditory cortex (for review, see Teki et al., 2012; Chauvigné et al., 2014; Merchant et al., 2015; Lusk et al., 2016; Petter et al., 2016; Braun Janzen and Thaut, 2018; Koshimori and Thaut, 2018). Specifically, cortico-cerebellar networks have been shown to be predominantly engaged in movement synchronization to externally cued stimuli (Buhusi and Meck, 2005; Brown et al., 2006; Del Olmo et al., 2007; Thaut et al., 2008, 2009; Witt et al., 2008; Manto et al., 2012; Chauvigné et al., 2014), whereas basal ganglia-thalamo-cortical networks seem particularly involved in beat-based timing and self-paced or internally driven rhythmic movements (Halsband et al., 1993; Cunnington et al., 1996; Rao et al., 1997; Grahn and Rowe, 2009, 2013). Furthermore, recently emerging evidence also indicate that auditory and motor areas have direct routes of communication at cortical level via the arcuate fascicle, a white matter fiber tract with direct projections from the auditory cortex to motor areas, including primary motor cortex and premotor cortex (Fernández-Miranda et al., 2015; Wang et al., 2016).

Another crucial aspect is that the functional and structural architecture of the auditory system is built to rapidly detect temporal patterns of periodicity in acoustic signals. There is considerable evidence that the temporal resolution of the auditory system is superior to other sensory modalities (e.g., Repp and Penel, 2002, 2004; Grondin and McAuley, 2009; Shelton and Kumar, 2010; Grahn et al., 2011; Stauffer et al., 2012; Ammirante et al., 2016). Recent electrophysiological research has demonstrated that the temporal information of acoustic signals is highly preserved at all levels of the auditory processing stream and elicit a periodic neural response at the exact same frequency of the stimuli (for review, see Nozaradan, 2014). Moreover, listening to auditory rhythmic stimuli primes the motor system, increasing the neural efficiency of the motor cortex through a process of auditory-motor entrainment (Crasta et al., 2018). That is, the firing rates of auditory neurons triggered by auditory rhythmic information, such as the beat of the music or a metronome, entrains the firing patterns of neurons in the motor cortex. The oscillatory coupling of neural impulses in the cortical loop between auditory and motor regions generates temporal predictions that are crucial for the perception of, and entrainment to, auditory rhythms (Large and Snyder, 2009; Fujioka et al., 2012; Large et al., 2015; Merchant et al., 2015; Ross et al., 2016, 2017; Morillon and Baillet, 2017). Therefore, the continuous time reference of the rhythmic auditory cues provides predictable feedforward information that allows movement anticipation and motor preparation (Thaut et al., 2015). Additionally, it has been shown that external rhythmic auditory input also changes the pattern of muscle activation through changes in corticospinal excitability (Thaut et al., 1992, 1999; Miller et al., 1996; Wilson and Davey, 2002; Stupacher et al., 2013), modulates beta (β) brain oscillations (Fujioka et al., 2012; Merchant et al., 2015; Ross et al., 2016, 2017), and promotes neural-plasticity (Luft et al., 2004). Collectively, these findings provide strong evidence of the neurobiological mechanisms underlying the effects of RAS on motor planning and execution.

The use of real-time movement information extends the benefits of discrete rhythmic auditory stimuli by adding an auditory component to the movement cycle either with natural movement sounds or movement sonification (Effenberg, 2005; Sigrist et al., 2013; Effenberg et al., 2016; Bevilacqua et al., 2016; Dyer et al., 2017a). Robust evidence suggests that merely listening to action-related sounds activates the neural processes necessary to produce those sounds (e.g., Aziz-Zadeh et al., 2004; Lewis et al., 2005; Pizzamiglio et al., 2005; Aziz-Zadeh et al., 2006; Gazzola et al., 2006; Caetano et al., 2007; Pazzaglia et al., 2008; Alaerts et al., 2009; Engel et al., 2009; Ticini et al., 2012; reviewed in Aglioti and Pazzaglia, 2010). Kohler et al. (2002) provided the first empirical evidence that premotor neurons in monkeys respond to the sound of a familiar action, expanding the notion that movements and their perceptual consequences are intrinsically coupled in the brain (Fadiga et al., 1995; Rizzolatti and Craighero, 2004; Schütz-Bosbach and Prinz, 2007; Rizzolatti and Sinigaglia, 2010). In humans, research shows that acoustic information are sufficient to evoke accurate representations of complex movements (Repp and Knoblich, 2004; van der Zwan et al., 2009; Lewis et al., 2011; Murgia et al., 2012a; Sevdalis and Keller, 2014; Kennel et al., 2014a; reviewed in Pizzera and Hohmann, 2015), activating superior and medial posterior temporal regions involved in human motion recognition (Bidet-Caulet et al., 2005; Baumann and Greenlee, 2006; Saarela and Hari, 2008; Scheef et al., 2009; Schmitz et al., 2013). Importantly, motor resonance is associated with and strengthened by one’s experience and familiarity with the actions observed/perceived, as demonstrated by studies comparing expert and novice responses to specific sports- or dance-related sounds (e.g., Agostini et al., 2004; Hohmann et al., 2011; Tomeo et al., 2012; Woods et al., 2014; Murgia et al., 2017). Further evidence of the role of learning and expertise has been provided by research showing that a network comprising areas such as dorsolateral and inferior frontal cortex (including Broca’s area), superior temporal gyrus, and motor areas including supplementary motor and premotor areas, is engaged when experienced musicians listen to well-rehearsed music (Haueisen and Knösche, 2001; Bangert et al., 2006; D’Ausilio et al., 2006; Harris and De Jong, 2014; see also Proverbio et al., 2014) or watch silent video recordings of known music pieces (Lotze et al., 2003; Hasegawa et al., 2004; Baumann et al., 2007; Bianco et al., 2016; reviewed in Maes et al., 2014; Novembre and Keller, 2014). Activation of this network was also found when non-musicians listened to a music piece they had learned to play after a short period of training (Lahav et al., 2007; see also Bangert and Altenmüller, 2003). These findings thus suggest that strong auditory-motor associations are developed during sound-making experiences, providing support for the use of real-time auditory feedback to enhance sensorimotor representations and facilitate movement (re)-acquisition.

It is also thought that the continuous availability of information provided by mapping different dynamic or kinematic movement parameters onto distinct sound components (e.g., pitch, loudness, rhythm, timbre) improves movement quality and motor (re)learning through the integration of multiple congruent perceptual streams (Scholz et al., 2015; Effenberg and Schmitz, 2018; Ghai et al., 2018c), resulting in a richer and more effective internal representation of the movement (Shams and Seitz, 2008; Wolpert et al., 2011; Schmitz et al., 2013; Effenberg et al., 2016). Furthermore, the availability of real-time auditory feedback also enhances online error-correction mechanisms (Dyer et al., 2015; Hossner et al., 2015; Sigrist et al., 2015; van Vugt and Tillmann, 2015), increases cognitive-emotional functioning (Van Vugt et al., 2014; Altenmüller and Schlaug, 2015; Sihvonen et al., 2017), and may supplement perceptual deficits (Tinazzi et al., 2002; van Vugt and Tillmann, 2015; Danna and Velay, 2017; Ghai et al., 2018c).

## Discussion

The studies here reviewed examined the relationship between sound and movement in the context of sports training and movement rehabilitation. Our narrative synthesis focused specifically on the literature regarding the effect of natural movement sounds, movement sonification, and rhythmic auditory information. The current state of knowledge here summarized provides promising evidence of the effect of auditory information on sporting performance and motor (re)learning.

The availability of auditory information in the form of natural sounds occurring as a byproduct of a movement or as additional real-time acoustic feedback driven by movement dynamic or kinematic parameters has significant implications for motor execution and control of skilled performances. The large body of research here reviewed indicates that auditory information provides crucial information about agency (Murgia et al., 2012a; Kennel et al., 2014a,b), movement control and timing (e.g., Chollet et al., 1988, 1992; Schaffert and Mattes, 2011, 2016; Sigrist et al., 2016; Schaffert et al., 2017), movement execution (e.g., Agostini et al., 2004; Konttinen et al., 2004; Baudry et al., 2006; Murgia et al., 2012b; Yang and Hunt, 2013, 2015; Kennel et al., 2015), and performance error/deviation (e.g., Höner et al., 2004; Godbout and Boyd, 2010; Wolf et al., 2011; Godbout et al., 2014; Ramezanzade et al., 2014; Tarnas and Schaffert, 2017). Behavioral data also suggest that the auditory-motor coupling generated during extensive training significantly interacts with athletes’ internal motor simulation (Murgia et al., 2012a; Kennel et al., 2014a,b; Pizzera et al., 2017), as shown by studies demonstrating that skilled athletes are able to extract highly specific information from action-related sounds (e.g., Roberts et al., 2005) and predict another person’s movements based on action prediction mechanisms (e.g., Cesari et al., 2014; Camponogara et al., 2017; Allerdissen et al., 2017). These findings corroborate a robust body of neuroimaging and neurophysiological studies indicating that the mirror neuron system and a widely distributed neuroanatomical network is involved in the processing of action sounds (e.g., Fadiga et al., 1995; Kohler et al., 2002; Aziz-Zadeh et al., 2004, 2006; Bidet-Caulet et al., 2005; Lewis et al., 2005; Pizzamiglio et al., 2005; Pazzaglia et al., 2008; Ticini et al., 2012; Schmitz et al., 2013).

Studies also demonstrated positive effects of auditory information on motor (re)learning in sports and rehabilitation. Research findings revealed that real-time auditory feedback facilitates learning and improves retention of new motor skills (e.g., Chollet et al., 1992; Konttinen et al., 2004; Baudry et al., 2006; Ramezanzade et al., 2014; Schaffert and Mattes, 2014; Pizzera et al., 2017). There is growing support for the application of movement sonification to increase upper-limb functions after stroke (e.g., Wallis et al., 2007; Immoos et al., 2013; Schmitz et al., 2014, 2018; Scholz et al., 2015, 2016; Ghai, 2018), and to improve gait in PD patients using, for instance, instrumented footwear (e.g., Batavia et al., 2001; Rodger et al., 2014; Horsak et al., 2016; Maculewicz et al., 2016; Gorgas et al., 2017). These sonification approaches rely on the transformation of dynamic and kinematic movement parameters onto distinct sound components (e.g., pitch, loudness, rhythm, timbre) to increase cross-modal stimulation (Scholz et al., 2015, 2016; Ghai et al., 2018c) and sensorimotor representation of the movement to be (re)learned (Shams and Seitz, 2008; Schmitz et al., 2013; Effenberg et al., 2016).

On the other hand, another line of clinical studies summarized in this review focuses primarily on the rhythmic patterns of sound, making use of metronome or beat-enhanced music to facilitate rehabilitation of intrinsically rhythmical movements (Thaut, 2005; Thaut and Hoemberg, 2014; Murgia et al., 2015; Ghai et al., 2018b). This robust body of research evidence indicates that RAS has immediate effects on gait velocity, step cadence, and stride length (e.g., Thaut et al., 1996; McIntosh et al., 1997; Freedland et al., 2002; Nieuwboer et al., 2007; Arias and Cudeiro, 2008, 2010; Hove et al., 2012; Song et al., 2015; Pau et al., 2016), reducing gait variability (Miller et al., 1996) and improving walking stability in PD (Thaut et al., 1999; Hausdorff et al., 2007; Hove et al., 2012) and stroke (Thaut et al., 1993, 1997, 2007; Hayden et al., 2009; for review see Thaut and Abiru, 2010; Hollands et al., 2012; Thaut and McIntosh, 2014; Nascimento et al., 2015; Yoo and Kim, 2016). Studies have also demonstrated that auditory cueing significantly improves upper-limb function after stroke by reducing movement variability and reliance on compensatory movements (e.g., Whitall et al., 2000; Thaut et al., 2002a,b; Luft et al., 2004; Jeong and Kim, 2007; Malcolm et al., 2009; Chen et al., 2016). It has been proposed that the continuous time reference provided by the rhythmic auditory cues facilitates movement retraining by priming the motor system, allowing movement anticipation and motor preparation (Thaut et al., 2015), and potentially bypassing damaged areas through the activation of alternative pathways (Hoemberg, 2005; Dalla Bella et al., 2017; Braunlich et al., 2018).

We also identified a small number of studies that evaluated other variables influencing the effect of auditory information on motor performance, such as physiological arousal and motivation (Murgia et al., 2012b; Bood et al., 2013; Immoos et al., 2013; Pugliese and Takala, 2015; Scholz et al., 2016; Sanderson and Hunt, 2016; Newbold et al., 2017). Murgia et al. (2012b) found that athletes’ maintained peak performance and reduced performance variability in trials where high-intensity sounds were presented during the pressing phase of weightlifting exercises, and Bood et al. (2013) reported changes in psychophysical and physiological outcome measures due to the motivational aspects of the stimuli during running. Novel therapeutic approaches, such as musical sonification (Scholz et al., 2016), also considered the motivational aspects of adding real-time auditory feedback to stimulate patients and improve treatment compliance, thus opening new avenues to systematically examine the role of physiological arousal, motivation, reward, and mood in larger clinical trials. The potential use of interactive sonification systems in sports and rehabilitation has motivated researchers and engineers to develop applications and system prototypes for exercise and physical activity, rehabilitation, and entertainment (e.g., Barrass et al., 2010; Lécuyer et al., 2011; Franco et al., 2013; Bruckner et al., 2014; Contreras Lopez et al., 2014; Pugliese and Takala, 2015; Newbold et al., 2017; see **Supplementary Material**). These studies explore a wide range of devices and applications where the playful character of music or the competitive component of sports has inspired new technology-enabled forms of play (e.g., exertion or computer games) and therapy. Future applications of this technology in sports, recreation, and rehabilitation are yet to be fully explored.

The large body of literature here reviewed clearly shows an emerging area of clinical and applied research. However, there are important research gaps that need to be addressed in future research. Firstly, there is a clear need to better understand what auditory components and amount of information are most relevant in motor training and rehabilitation. This is not trivial, particularly in sonification applications, as research suggests that an overload of auditory information has detrimental effects on task performance (e.g., Wolf et al., 2011) and that task-irrelevant auditory stimuli are strong distractors (Parmentier, 2014). The use of meaningful auditory information is, therefore, determinant for the user’s experience (Effenberg et al., 2016; Dyer et al., 2017b) and needs to be considered in a clear framework for sonification mapping derived from a better understanding of the processes underlying motor learning/control from a basic research perspective (Dyer et al., 2015). Research in the field of auditory information processing has great potential to promote active crosstalk between basic and applied research, with findings generated in the laboratory providing insights for the application in real-life situations, that being in sports training or therapy and rehabilitation, and vice-versa. Secondly, we have identified few studies using natural movement sounds or sonification in elite or high-performance sports. A challenge for future investigations is to evaluate novel applications in ecologically valid and real-life situations that closely resemble the athlete’s movement technique and training conditions in order to better identify what type of information is most relevant and improve equipment setup, thus acquiring more reliable results. This depends directly on the development of procedures that are feasible for the systematic use in daily training. In addition, future research should also consider the way in which auditory information is presented to athletes and patients (loudspeaker vs. earplugs) in order to avoid, for instance, perceptual overload, and to ensure that the feedback information is properly delivered. From the clinical perspective, although there is growing attention on the application of sonification systems to improve motor function in PD and post-stroke, we have identified a relatively small number of controlled trials, revealing the need to further examine the effectiveness and feasibility of sonification methods and devices in larger controlled clinical studies.

## Conclusion

This review examined the relationship between sound and movement in the context of sports training and movement rehabilitation. The findings here summarized provide evidence of the effect of natural movement sounds, movement sonification, and rhythmic auditory information on sporting performance and motor (re)learning. This emerging area of clinical and applied research demonstrates large underutilized potential, warranting further investigation of the promising application of auditory feedback information in sports and rehabilitation.

## Author Contributions

All authors listed have made a substantial intellectual contribution to the work and approved it for publication. NS conceived and drafted the first version of the manuscript. TBJ contributed to the writing and revision of the manuscript. MT and KM supervised and revised the manuscript.

## Conflict of Interest Statement

The authors declare that the research was conducted in the absence of any commercial or financial relationships that could be construed as a potential conflict of interest.
