# Supplementary material for: A Review on the Relationship Between Sound and Movement in Sports and Rehabilitation
Source: Front Psychol. 2019 Feb 12;10:244. doi: 10.3389/fpsyg.2019.00244 (PMC6379478; doi:10.3389/fpsyg.2019.00244)
Supplement: Supplementary file 1 [file Table_1.pdf]

Supplementary Material

A review on the relationship between sound and movement in sports and rehabilitation

Nina Schaffert [1*], Thenille Braun Janzen [2], Klaus Mattes [1], Michael H. Thaut [2]

[1]Department of Movement and Training Science, Institute for Human Movement Science, University of Hamburg, Hamburg, Germany

[2]Music and Health Science Research Collaboratory, Faculty of Music, University of Toronto, Ontario, Canada

*** Correspondence:** Dr. Nina Schaffert: nina.schaffert@uni-hamburg.de

Supplementary Table 1. Overview of sonification systems and applications in sports.

KEY TOPIC 1<br>SPORT			
**MOVEMENT SONIFICATION**			
**Reference**	**Type of Sports/ Activity**	**Technology/ Measurements**	**Aim, Main Findings/ Conclusions**
Almquist Gref et al. 2016	Manual Wheelchair operation	System prototype comprised of a wheelchair equipped with three passive OptiTrack Prime 41 motion capture markers, a computer running Pure Data, and eight speakers set up in an octagon shape.	This pilot study implemented a mapping approach where key parameters of manual wheelchair maneuvering were directly mapped to different sound models. The study concluded that sonification of manual wheelchair movements with direct mapping from movement parameters does not seem to produce meaningful feedback for all types of manual wheelchair operation. The authors, therefore, suggested that the level of difficulty of the exercise and the perception of synchronization between movement and sound in the system are crucial in these types of applications.
Anlauff et al. 2013	Slackline	Auditory feedback system sensing the user's posture with a Microsoft Kinect.	The study presented a system prototype that provides augmented feedback for learning single-legged stance on a slackline. The aim of the system was to support beginner slackline stance practice with augmented auditory feedback on two key posture parameters: leg bent and upper body stability. The study did not present empirical results.
Avissar et al. 2013	Balance and posture control	Interactive audio game app prototype that assesses posture control.	This paper introduced a prototype for an accessible virtual gaming interface that uses sway-detection algorithm,

			music, and pitch as positive reinforcement in the accomplishment of target postures. This system is yet to be implemented in a study validating its effectiveness as a therapeutic tool and its entertainment value as a game.
Barrass et al. 2010	Recreational Sports	Sweatsonics technology – a mobile device that synthesizes six different interactive sonifications of acceleration.	This pilot study developed a 'technology probe' method and device to investigate preferences between six different interactive sonifications in recreational sporting activities. In initial testing, the system was used to capture acceleration data from participants engaged in outdoor physical activities.
Baudry et al. 2006	Gymnastics	Sonification system comprised of a two-piece device placed on the upper back and knee connected by a cable. Increased cable tension delivered an auditory signal indicating hip flexion greater than 20 degrees.	The study tested a sonification device that was designed to deliver an auditory alarm whenever hip flexion was greater than 20 degrees. Gymnasts in the experimental group wore the sonification device while training circle movements on a pommel horse, whereas athletes in the control group did not receive auditory feedback. Results indicated that athletes in the experimental group significantly improved hip flexion after a 2-week period in comparison to the control group. Findings also suggested retention of the benefits after a further 2-week training without the device.
Bolibar and Bresin, 2012	Long-distance running	Microsoft Kinect camera in combination with the OpenNI system capable of analyzing the vertical displacement, torso tilt, and step distance of the athlete.	A preliminary study testing a sonification system to improve running technique by providing an auditory signal whenever there was a posture deviation. The article discussed some challenges faced during the implementation of the system

			and possible set up solutions. No empirical data was presented.
Bood et al. 2013	Running	Treadmill running in the laboratory.	The study tested the relative effects of acoustic stimuli on running performance. Runners were divided into three groups: in one condition participants listened to metronome beeps matching their running cadence, whereas the second group listened to motivational music matched to the participant's cadence. Belt speed was progressively increased every 30 seconds from 9 km/h upwards in a 5-minute protocol. The authors observed that psychophysical and physiological outcome measures were more affected by the motivational aspects of the music, whereas running performance was most efficient in the metronome condition as the metronome beats helped participants to maintain a consistent and optimal running pace.
Bovermann et al. 2007	Juggling	JUGGLING SOUNDS is a system for real-time auditory monitoring of juggling patterns consisting of data acquisition via motion tracking and sonification via customized TUIO server.	The study introduced a new approach for auditory monitoring of real-time data acquired by motion tracking of juggling clubs. The system used both direct mappings of low-level feature-streams and detected events for the sound synthesis, capturing swinging, rotation, and juggling movements.
Boyd and Godbout, 2012	Walking and Running	A system capable of transforming joint-angle signals in auditory information.	The paper presented a sonification method that uses joint-angle signals produced by a motion capture system (Vicon) to generate drumbeat sounds in synchrony to the individuals' footfalls during walking and running. The method estimates

			the phase of a periodic, multidimensional model to match data observed from a moving person using an optimization algorithm. Preliminary results with publicly available motion capture database suggested the feasibility of the method proposed.
Boyd et al. 2012	Speed Skating	A robot vision system that captures the full-body motion of athletes during real training situations.	This preliminary study tested a robotic system that follows a speed skater on the ice with a Kinect in order to capture full-body motion in situ. Although the researchers encountered some challenges implementing the system due to limitations of the Kinect, the system seemed to produce valuable data to coaches and athletes.
Cesarini et al. 2014a	Swimming	A system utilizing 4 differential pressure transducers attached between the fingers of each hand to measure hydrodynamic pressure.	The preliminary study introduced a system for the recording and presentation of real-time sonification of hydrodynamic pressure during swimming in water. The system was designed to acquire pressure data from the swimmer's hands, process it, and transform it in real-time auditory biofeedback, which is then presented to swimmers through in-ear underwater earphones. The publication discussed the system setup.
Cesarini et al. 2014b	Rowing	The Accrow device (BeSB GmbH and University of Hamburg) consists of a MEMS (Micro-Electro-Mechanical Systems) acceleration sensor and a GPS receiver. This system was used to measure rowing acceleration and velocity.	The study presented a sonification platform (AccrowLive 2.0) designed to present real-time visual and acoustic feedback about rowing acceleration and velocity during on-water rowing training. This multi-platform software solution may be used by coaches during on-water training as well as for

			later off-line analysis of athletes' performance.
Chollet et al. 1988	Swimming	Sonification system designed to measure hydrodynamic pressure at hand paddles and deliver real-time auditory information.	The study tested the application of a sonification system to measure hydrodynamic pressure exerted by the athlete's hand during the propulsive action in crawl swimming. The system transformed the information captured into a real-time audible sound of equal amplitude and mapped to tone pitch (higher pressure was audible as higher tone pitch). Testing on a single training session indicated that the availability of concurrent auditory feedback increased consistency of swimming velocity and maintenance of stroke count.
Chollet et al. 1992	Swimming	Sonification system designed to measure and map hand hydrodynamic pressure and waist velocity onto real-time auditory information.	This study extended the work of Chollet et al. 1988 and evaluated the short- and long-term effects of real-time auditory information on swimmers' performance. The results indicated that athletes who trained with sonification showed improved movement coordination and swimming velocity after 5 days of training. It was also found that the improvements were maintained at a retention assessment after 10 days of training without sonification.
Dubus and Bresin, 2010	Rowing	Sonification system that consists of a GPS receiver embedded in a smartphone taken onboard and an external accelerometer.	This preliminary study tested two interactive sonification system designed to measure boat position, mean velocity and acceleration. One of the systems used pure tones of variable frequency, and the second sonification system made use of the MIDI synthesizer built in a mobile phone to generate musical sounds. The results from

			preliminary tests showed the potential of parameter-mapping sonification to provide meaningful sound feedback, however, the authors acknowledge that the auditory display did not meet the aesthetic requirements for a final version of a sonification system, requiring further improvement.
Eriksson and Bresin, 2010	Running	A system based on an accelerometer sending data to a mobile phone to provide feedback on the runner's vertical displacement of the center of mass.	A pilot study testing a platform to improve running mechanics with an interactive sonification system. The results showed a promising application of this non-obtrusive portable platform.
Godbout and Boyd, 2010	Speed-skating	The sonification system comprised of a sensor placed between the toe and shin of the skater. The system measured the athlete's ankle extension during a routine movement and provided corrective sonic feedback based on a model.	A case study using a sonification system to provide real-time corrective sonic feedback. The system measured the athlete's ankle extension during skating crossover strides and matched the real-time information with that of a model. The continuous sonic information allowed the athlete to make adjustments based on the stride model, thus improving movement quality. Preliminary tests demonstrated the feasibility of this sonification system in routine training.
Godbout et al. 2014	Speed-skating and running	Sonification system based on the Armour39 Under Armour wearable motion and heart-rate sensor.	This case study tested a sonification system that exploits the periodicity of human motion to produce synchronized acoustic rhythms that convey timing information to the athlete. For speed-skating, the authors sonified the basic movement as a rhythmic arpeggio synchronized with the stride and transposed the arpeggio to different chords corresponding to the stride type. For running, short tones were played in synchrony with the footfalls, alternating in pitch between left and right feet. This

			study showed that the sonification of athletic movements from wearable, consumer sensors, and mobile devices is possible.
Hasegawa et al. 2012	Skiing	Sonification system that measures the position of the center of gravity with force sensors and maps this information on to sound feedbacks.	This preliminary study tested a device prototype that provides real-time simple sound feedbacks of the center of gravity of the skier. The study found that sonification of the center of gravity was an effective method to improve body position.
Hermann et al. 2012	Swimming	Sensor system attached to the arm of the swimmer.	This paper introduced a sonification system for pressure sensor data measured while executing crawl stroke swimming. Hydrodynamic pressure measured at 5 positions along one arm was mapped into a complex sonic rhythmical melody whereby athletes could identify differences and variations between patterns. The methodology may serve as the basis for future real-time sonification to be developed.
Hermann and Zehe, 2011	Aerobics	A portable system to measure arm and leg movements using wearable motion sensors (goniometers) that send digitized data via Bluetooth to a computer equipped with Supercollider program to render data sonifications either in real-time or from pre-recorded data.	The publication discussed initial testing of two potential sonification approaches for the sonic representation of coordinated body movements for a single performer during aerobics.
Höner et al. 2004	Handball	Sonification system for analyzing tactical behavior of sports teams.	This pilot study presented a sonification system designed to support tactical training by tracking the position of the players and the ball and comparing the data obtained to the tactical expectations. The

			system was unable to display real-time data for technical reasons requiring further developments.
Hummel et al. 2010	German wheel	The system uses a magnetometer to collect data about the motion of the wheel.	This abstract introduced the design, implementation, and evaluation of a sonification system that presents real-time auditory feedback of the rolling motion of the German wheel. The study was carried out with a group of seven novices and four experts and showed a positive influence of the event-based sonification approach on the experts' performance of a given task.
Kirby, 2009	Skiing	Real-time performance measurement and feedback system for training alpine skiers using Optical Navigation Technology.	The preliminary study tested a new method for measuring and providing auditory information about skiers' lateral displacement in real-time. The effectiveness of the system was assessed through interviews and questionnaires where the majority of athletes and coaches perceived the benefits of real-time feedback after a two-hour session. However, the study did not examine whether the system had any effects on the athletes' actual performance.
Kleiman-Weiner and Berger, 2006	Golf	Sonification model that creates an auditory mapping of the velocity of the club head and the relative rotation of shoulders with respect to the hips.	The study presented a model for multidimensional auditory display of physical motion by mapping club head velocity and relative shoulder rotation on to phoneme-based sonification. The purpose of the model is to facilitate learning, but no testing results assessing the effectiveness of the model were presented in the paper.

Konttinen et al. 2004	Precision shooting	The system comprises of an optical transmitter/receiver mounter of the rifle capable of capturing the on-target trajectory of rifle alignment, measuring the distance between the shooter's aiming point and the center of the target.	The study evaluated a sonification system designed to capture rifle alignment in relation to a target. The trajectory data is sonified providing auditory information that varies with the location of the aiming mark on the target area. The results demonstrated that non-elite shooters who trained with sonification for 4 weeks displayed more accurate shooting performance than those who did not receive auditory information during training. It was also found that the improvements were maintained on retention assessments conducted after 10 and 40 days post-training.
Murgia et al. 2012b	Weightlifting		The study tested whether the presence of an auditory stimulus during exercise execution would affect power exerted during the lifting. The stimulus consisted of a low-intensity sound corresponding to the down phase of the exercise and a high-intensity sound associated to the pressing phase. The results indicated that the presentation of the external auditory feedback increased the average exertion of power during weightlifting, suggesting that the information was used by athletes to maintain an adequate level of activation and reduce performance variability, thus optimizing sports performance.
Newbold et al. 2017	Squat jumps	Musically-informed sonification of movement using the squat-down exercise.	This study discussed the motivational aspects of music to support people who struggle with physical activity. Initial test

			results showed that musical expectancy impacted people's perception of their own movement in terms of reward, motivation and movement behavior.
Nylander et al. 2014	Golf	SwingSound 9D movement sensor that provides real-time feedback on movement acceleration	The paper introduced a sonification system (SwingSound) that creates an audio mirror of the golf swing in real time.
Powell and Lumsden, 2015	Motorsport	Tonal audio feedback representing G-force data during driving	This paper explored the development and evaluation of a novel real-time auditory display system for accelerated racing driver skills acquisition. Real-time audio feedback representing lateral G-force (a proxy for tire slip) is delivered to one ear whilst a target lateral G-force value representing the 'limit' of the car is panned to the driver's other ear. The system was tested in a preliminary study in a driving simulator environment. Initial results indicated that driver confidence was enhanced when audio feedback was presented.
Pugliese and Takala, 2015	Elastic Trampoline	Interactive sonification system based on motion tracking with a depth camera and audio-based contact sensing between the feet and the trampoline.	The paper examined the influence of auditory augmentation feedback to increase performance and enjoyment during jumping exercises on an elastic trampoline. The sound generated by the jumps was inspired by iconic jumping sounds from games resembling a spring, with a frequency sweep from low to high pitch. Results showed that auditory feedback was associated with higher jumps and that sonifications with body-controlled pitch induced a more engaging and pleasant

			experience over the fixed frequency condition.
Ramezanzade et al. 2014	Basketball	A sonification modeling system that sonified the angular speed of a player's elbow (model) based on data extracted by a motion analysis device.	The study tested the effect of audiovisual integration on accuracy and learning of the jump shot in basketball. A group of novice participants received only visual information from a model (professional player) while the experimental group also received auditory information provided by a sonification system. Preliminary findings suggested that the audiovisual group outperformed the visual group in both the acquisition and retention tests, suggesting that auditory information facilitated the learning of a new motor skill.
Sanderson and Hunt, 2016	Running	A mobile-based application that uses sonification of heart rate data to provide real-time, audible feedback to athletes.	The study tested a sonification system that aimed to improve the efficiency of heart-rate-zone based training amongst runners by combining the motivational benefits of music with parameter mapping sonification techniques. Initial testing results showed that the system was a helpful training aid to assist runners to successfully follow a predefined intensity based training plan.
Schaffert et al. 2010	Rowing	Sofirow - an acoustic feedback system for on-water rowing training that measures propulsive boat acceleration with a micro-electro-mechanical (MEMS) acceleration sensor and boat velocity with GPS, and converts this information into acoustic information sonification of the boat acceleration.	This article presented the initial test results of the effectiveness of a sonification system – Sofirow. The sonified boat motion changed as a function of the acceleration trace meaning that athletes perceived an increasing tone pitch the more the boat was accelerated. Preliminary results demonstrated the successful implementation of this system in elite rowing, and that sonification increased mean

			boat velocity and increased distance traveled per stroke.
Schaffert and Mattes, 2011	Rowing	Sofirow	This article presented the design and development of the acoustic feedback system Sofirow and preliminary test results, which confirmed previous pilot findings (Schaffert et al., 2010).
Schaffert et al. 2011	Rowing	Sofirow	This study examined the potential of acoustic feedback information to represent boat motion in rowing and its implementation online for the training of elite rowers in on-water training conditions. Results showed that the acoustic feedback helped the athletes to adjust their rowing strokes with an increase in the mean boat velocity and increased distance traveled per stroke. The auditory feedback also helped crew synchronization.
Schaffert and Mattes, 2014	Rowing	Sofirow	This study tested the immediate effects of acoustic feedback on mean boat speed during on-water rowing training with elite athletes. The boat's acceleration-time trace was converted online into acoustic feedback and presented to the athletes via speakers. Athlete's rowing technique was assessed before, during and after a 2-week period. The study demonstrated that acoustic feedback significantly improved mean boat speed and that the benefits of sonification were retained at the follow-up assessment.
Schaffert and Mattes, 2015a	Para-Rowing	Sofirow	This study examined the effects of auditory feedback on mean boat speed during on-water training of visually impaired

			athletes. The sonification system converted boat acceleration-time traces into acoustic feedback presented via speakers during rowing. Results demonstrated that the sonification system enhanced boat speed in relation to baseline and improved the timing structure of rowing cycles by an extended time of positive acceleration. Moreover, the system provided access to biomechanical information for visually impaired athletes.
Schaffert and Mattes, 2015b	Rowing	Sofirow	The study surveyed elite athletes from 2009 to 2013 to assess their perception regarding the different criteria and requirements for an auditory display to be used in a moving context by considering distinct mapping strategies. The results indicated that athletes perceived that changes in pitch within the rowing cycle during propulsive-critical phases best represented the movement and that auditory feedback increased crew synchronization.
Schaffert and Mattes, 2016	Rowing	Sofirow	The study investigated the effects of acoustic feedback on boat speed, rowing technique, and crew synchronization in elite junior rowing. The study confirmed previous test results and found that parameters of crew synchronization showed reduced ranges for the time during drive and recovery phase, rhythm quotient, and handle speed during the drive phase with acoustic feedback compared to baseline.

Schaffert et al. 2017	Cycling	Interactive sonification system (Wattbike ergometer)	This preliminary study presented an interactive sonification system designed to improve pedaling technique and pedal force efficacy during cycling using the Wattbike ergometer. Initial testing showed that the sonification system improved pedaling cycle and force symmetry, and allowed the cyclist to perceive fluctuations in forces applied on the pedals and adapt muscle activation patterns accordingly.
Sigrist et al. 2016	Cycling	Sonification system that mapped crank moment to auditory feedback.	This pilot study investigated the effects of sonification during complex bipedal pushing-pulling action on a cycling ergometer. The crank moment was linearly mapped to the frequency of a violin sound so that higher crank moments resulted in higher frequencies. The goal of the participants' task was to match the frequency from the left earphone (representing the own moment applied with the left leg) to the frequency heard in the right earphone (representing the reference moment) using the frequency differential as feedback. Preliminary results showed inconsistent effects on learning, which were attributed to an inappropriate sonification design, short training sessions, or the high task complexity.
Stienstra et al. 2011	Speed-skating	Sonification system comprised of two speed-skates and central processor MAX5 (100 Hz)	This case study presented a sonification system (Augmented Speed-skate Experience) that was designed to present feedback on skating technique to a professional athlete. The study results indicated that the movement sonification was well received by the athlete, but further research is needed to test

			its applications in a larger sample.
Tarnas and Schaffert, 2017	Sailing	Smartphone application for course deviation using data from GPS and geometrical calculation.	The application was developed to provide sailors with auditory information about deviations from an optimal course covering tactical situations such as sailing upwind – tacking and sailing downwind. The sound was generated either as a pure tone, speech, repeated sound or personal setting. Pilot tests with professional sailors showed that the application audibly reflected the course of the vessel and was particularly helpful to get information for making a tactical decision for the last tack before rounding the mark.
Wolf et al. 2011	Rowing	Error sonification system using a virtual display of the oar blade on a large screen; oar motion was captured in real-time by an optoelectronic motion tracking system.	The study evaluated the potential of error sonification to enhance the learning of a rowing-type motor task. Deviations to the horizontal oar angle were mapped to stereo-balance, deviations to the vertical oar angle to pitch, and wrong oar-blade orientation was mapped to a raspy sawtooth wave. Results indicated that the experimental group was able to use the auditory feedback to reduce spatial and temporal errors during the training. However, they were not able to improve their performance in the retention tests. The authors also indicated that the auditory feedback remained unfamiliar during the first half of all training sessions, which suggested that the auditory feedback was very demanding, hindering learning of the task.

Yang and Hunt, 2013, 2015	Muscular training (biceps curl)	A real-time sonification system that uses a parameter mapping method based on exercise information collected from a muscle sensor and Kinect camera.	A preliminary study investigated whether movement sonification would improve movement (bicep curl) technique. Initial results indicated that the sonification system provided relevant information about the timing of movement, helping to improve movement pacing and exercise metrics.

Supplementary Table 2. Overview of sonification and auditory feedback systems and their application in therapy and rehabilitation.

KEY TOPIC 2<br>REHABILITATION			
**MOVEMENT SONIFICATION**			
**Reference**	**Therapy Target**	**Technology/ Measurements**	**Aim, Main Findings/ Conclusions**
Batavia et al. 2001	Gait and standing symmetry	Augmented auditory feedback device triggered by weight applied to a thin membrane switch mini-buzzer that fits inside a patient's shoe without altering the heel height.	A case study evaluating a system prototype consisting of a sensor that provides acoustic information (e.g., buzzer sounds) whenever the patient places sufficient weight on the heel, providing information about the occurrence, duration, and distribution of weight bearing. Initial test results indicated that the augmented auditory feedback device provided important information that led to rapid improvement in balance and gait, warranting further investigations in larger controlled studies.
Bresin et al. 2010	Walking	Sensored-shoes capturing foot pressure with Interlink 400 force sensing resistors, 2 per insole (front and rear). Foot pressure is used for informing different real-time synthesis models, whose output feeds the shoe-mounted haptic actuators and loudspeakers.	This preliminary study evaluated a system prototype for sonification of footsteps and examined the impact of ground texture sound on gait parameters such as heel-to-toe and heel-to-heel inter-onset intervals. The results showed some trends of a possible interaction between the quality of the auditory feedback and walking patterns.
Bruckner et al. 2014	Upper limb; Stroke	Smartphone-based hardware platform for interactive, low latency movement sonification based on inertial measurement units.	The study evaluated a sonification prototype that displays wrist position in relation to the patient’s shoulder joint based on customizable parameter mappings. The sonification demonstrator based on consumer electronic device could be used in studies for determining the benefits of movement sonification in stroke rehabilitation and sports training sessions.
Chen et al. 2006	Upper limb; Healthy individuals	A system based on virtual reality and movement sonification	This pilot study tested the feasibility of a therapeutic system involving virtual reality and movement sonification. Patients’ arm

		to provide multimodal information and facilitate recovery of upper limb function.	and torso movements were captured through motion capture markers and motion data during reaching and grasping tasks. Movement data were mapped onto different auditory/musical components. Pilot tests with healthy individuals showed that participants were able to use the auditory information to improve spatial accuracy and movement flow, warranting further tests in controlled clinical studies.
Chiari et al. 2005	Balance; Healthy individuals	Customized audio-biofeedback (ABF) device with accelerometers and a force plate to estimate body sway by means of the center of pressure data.	This preliminary study tested a prototype sonification system for balance improvement based on trunk kinematic information. The system converts real-time 2D horizontal trunk accelerations into a stereo sound by modulating its frequency, level, and left/right balance. Preliminary results with healthy subjects showed improved balance, suggesting that this device may be a useful tool for rehabilitation.
Contreras Lopez et al. 2014	Gait training; Parkinson's Disease	External portable device and smartphone application (Listenmee) delivering auditory rhythmic cues matched to the patient's gait cadence.	The preliminary study tested the effects of a sonification system on gait. PD patients were first asked to walk at their fastest speed along a 7 meters walkway (baseline) and then to walk the same distance trying to synchronize each step with the auditory tones provided by the sonification device. Results of a single session indicated that gait velocity, cadence and stride length improved when the auditory information was present.
Dailly et al. 2012	Upper extremities; Healthy individuals	Error sonification system combined with music for upper limb training.	The purpose of this study was to test whether simple error sonification combined with music would help healthy individuals to learn a planar reaching movement more effectively than when receiving no feedback. The error-sonification feedback group received audio feedback during training, provided by music and white noise. In contrast, the control group just listened to background music during training and did not receive any feedback about performance. This study showed that a concurrent error sonification could assist unimpaired subjects to learn new movements.

Dozza et al. 2005	Balance; Healthy individuals	Audio-biofeedback system that provides participants with pitch and volume coding of body sway.	The study tested the effectiveness of an audio-biofeedback system prototype to reduce postural sway in stance. Healthy individuals performed standing tasks on a foam surface with eyes closed and received auditory information that varied in pitch, volume and left/right balance to represent trunk acceleration. Findings suggested that the availability of continuous auditory information significantly increased postural stability.
Dozza et al. 2007	Balance; Vestibular loss	Audio-biofeedback system that provides participants with pitch and volume coding of body sway.	The study extended previous tests (2005) and evaluated the effectiveness of an audio-biofeedback system to reduce postural sway in stance in both healthy controls and subjects with bilateral vestibular loss. Results indicated that the feedback system was effective in reducing body sway of the patients when the environment provided limited visual and somatosensory information.
Fischer et al. 2017	Walking	Auditory feedback system SONIGait consisting of 2 shoe insoles equipped with 7 force sensors located below the heel, at the lateral side and at the base of the metatarsal joints.	The study tested the effectiveness of the system in providing enhanced, real-time feedback during the stance phase of gait on healthy elderly participants. Application of the system resulted in altered spatiotemporal gait parameters.
Franco et al. 2013	Balance; Healthy individuals	Smartphone application (iBalance-ABF) equipped inertial sensors to estimate 3D angulation of the trunk to improve balance.	The study proposed the implementation of a smartphone application to monitor trunk angular evolution during bipedal stance to help users improve balance through a configurable and integrated auditory-biofeedback loop. Initial testing with healthy individuals suggested that the system promotes an immediate improvement of the control of bipedal posture, requiring further research to examine the feasibility and effectiveness of this application in rehabilitation.
Fujii et al. 2016	Upper limb; Healthy individuals	The system comprised of three goniometer sensors to collect	The study tested whether augmented auditory feedback would facilitate motor learning of a target-reaching task. The

		elbow, shoulder and trunk information during a reaching task.	system provided a pure tone that varied in intensity in relation to the performance error. Healthy participants learned a novel target joint coordination pattern. One group of participants received feedback on all trials whereas the second group received feedback on 50% of trials. Results showed that the group that received feedback on 100% of trials showed a significantly smaller root-mean-squared error at both immediate and delayed retention tests, suggesting that the presence of auditory feedback during the acquisition phase of a novel test significantly enhanced the retention of this skill.
Ghai et al. 2018d	Lower limb; Healthy individuals	The sonification system comprised of seven inertial sensors placed on the participant's dominant leg. Knee joint angle and angular velocity data were mapped onto pitch and amplitude of the auditory feedback, respectively.	The study tested the effect of auditory information to increase proprioception. For that, healthy participants performed an active knee re-positioning task that involved repeatedly placing the knee of their dominant leg at a pre-established target angle. Knee joint angle and angular velocity data were mapped onto pitch and amplitude, respectively. Findings suggested that knee re-positioning accuracy increased significantly with the concurrent presentation of real-time auditory feedback.<br>In a second experiment, researchers introduced subtle step-wise pitch modulations in the auditory feedback and found that participants tended to modulate their knee movements in the opposite direction of the transposition.
Gorgas et al. 2017	Gait; Parkinson's Disease	Foot pressure data were recorded and continuously sonified by an instrumented shoe insole device (SONIGait) during walking.	This pilot study tested the effectiveness of SONIGait on gait parameters in PD patients. A sonification model based on musical notes was used to sonify the individual walking characteristics. Results indicated that a five-minute practice phase with sonification led to an increase in gait velocity and cadence, which the authors attributed to a probable increase of attentional demands when practicing with SONIGait.
Horsak et al. 2016	Gait training; Parkinson's Disease	System prototype that consists of a pair of wireless sensor insoles instrumented with	This study evaluated a gait sonification system prototype. Preliminary testing on a small study sample indicated that the patients decreased cadence and presented

		force-sensors for real-time data transmission and acquisition on a mobile client (SONIGait).	changes in gait velocity when walking with sonification.
Immoos et al. 2013	Upper extremities; Stroke	Error sonification system in combination with music to improve motivation during repetitive paretic arm training tasks.	A pilot study testing the effects of error sonification. During ten training sessions, stroke participants were asked to complete tracing tasks while listening to their favorite music in the background or receiving error feedback. The study found that both groups (music background vs. music + sonification) improved upper limb mobility, thus, limited conclusions can be made.
Maculewicz et al. 2016	Gait;	Sonification system that detects user's footsteps and provides interactive real-time feedback or suggests a pace using a synthetic walking sound or vibration.	The study investigated the impact of auditory and haptic feedback on walking tempo. The results showed that participants synchronized equally well with the tempo with either audio or haptic cues, but multimodal audio–haptic feedback provided the most natural feeling. These results have implications for the design of interactive entertainment or therapeutic applications.
Maulucci and Eckhouse, 2001	Upper extremities; Chronic stroke	A system (hard- and software) and feedback (visual and auditory) algorithms were developed. Also, twenty reach parameters were defined and analyzed for differences between training sessions, between adaptation and learning trials, and among three targets.	The study aimed to enhance the motor recovery of patients in the chronic stroke period by means of upper extremity trajectory modification through real-time auditory feedback. Two groups of patients in the chronic stroke period were trained on the system, one with practice and feedback and one with practice alone. The results demonstrated that modification of the reach trajectory can be accomplished during the chronic stroke period either with practice alone or through practice accompanied by auditory feedback. It was concluded, that improved path performance requires auditory feedback training and cannot be achieved by practice alone.
Matsubara et al., 2012	Multiple muscular activities	A real-time sonification system using EMG, and three sonification methods representing the data in pitch, timbre, and the	The study reported preliminary tests of a real-time sonification system using EMG. Participants were asked to identify the timing and order of the auditory information presented, and answer a questionnaire about the subjective comprehensibility and

		combination of polyphonic timbre and loudness was developed.	preferences. Results of the listening tests showed that participants were able to accurately identify the information conveyed by the auditory signals and that there was not a direct relationship between the task performance accuracy and the preference of sound.
Mezzarobba et al. 2018	Freezing of gait; Parkinson's Disease	Action observation plus sonification of walking kinematics (pelvis movement velocity, and inferior limbs velocity) gathered via the motion-capture system.	The study aimed to test the effects of a new experimental protocol based on action observation plus sonification. Participants watched videos showing gait-related gestures while simultaneously listening to the sonification of the kinematics of each gesture. Patients then imitated the movements shown. The training protocol was completed twice a week for a total of 8 weeks. Assessments conducted after the intervention and 3 months after the treatment suggested that the multisensory treatment significantly reduced the number of episodes and duration of freezing, facilitating the priming effect generated by action observation. It was concluded, that this approach could help PD patients with freezing of gait to relearn gait movements, to reduce freezing episodes and that these effects could be prolonged over time.
Miyake, 2009	Gait	The system consists of a PC (placed in a waist pouch) that simulates the virtual walking robot, foot (acceleration) sensors (attached on both ankles) for detecting footsteps, and headphones for the sound stimuli.	The study aimed to investigate interpersonal synchronization in cooperative walking whereby participants walked with a virtual partner generating a rhythmic sound corresponding to the timing of the footsteps. Results demonstrated that the walking rhythms from the participant and the virtual partner mutually adapted after the start of the interaction, and a stable synchronization was generated automatically.
Pauletto and Hunt, 2006	Osteoarthritis of the knee	Real-time sonification of electromyographical (EMG) data acquired from EMG sensors connected to an analog-to-digital converter and a computer running the sound mapping software.	The paper described a sonification system to display EMG data gathered in real-time. The sonification uses amplitude modulation to create timbres that portray six channels of data. Results showed that some characteristics of the sound portrayed specific information about the data and/or the patient. For instance, the "roughness" of the sound's timbre was found to be

			correlated to the age of the patients. Overall, the study found that the sonification was considered to be appropriate to represent EMG data, i.e. muscle movement.
Robertson et al. 2009	Upper extremities; Stroke	Kinematic data derived from a Polhemus sensor fixed to the hand of the patient were processed online to modulate the auditory feedback provided by headphones.	The study examined the effects of two types of auditory feedback on movement kinematic during hand trajectory during a reaching task. Patients performed a task that involved reciprocal pointing to 9 targets in three feedback conditions: no feedback, simple feedback (volume increased as the hand approached the target), and spatial feedback (sound volume simulated binaural spatial cues with interaural differences). The study found no differences between the two auditory feedback conditions on movement kinematic. However, findings showed significant kinematics differences between patients with right or left hemisphere damage, and that the effect of auditory feedback differed according to the lesion side - with additional feedback benefiting only patients with right hemisphere damage.
Rodger et al. 2014	Gait; Parkinson's Disease	A computational audio synthesizing system to create artificial gravel walking sounds using an existing gravel sound synthesis program adapted to use an approximation of the ground-reaction force (GRF) between the foot and the gravel surface as input control data for the sound synthesis.	This pilot study tested two sonification approaches: one used ground-reaction force recordings to synthesize the sounds of footsteps on gravel with specific step lengths and durations, and the second approach involved real-time sonification of the swing-phase of gait using motion-capture data to control a sound synthesis engine. In the gravel sound condition, the participants were asked to try to imitate the sound they heard by walking in a way that would reproduce this sound if they were walking on a gravel path. Results suggested that both methods can be effective in reducing gait variability in PD patients, however further testing with larger patient samples is needed.
Schedel et al. 2016	Parkinson's Disease		This preliminary study tested whether people with PD were able to perceive complex auditory cues. For that, distortions relating to rhythm, timbre and white noise were superimposed on commercial music. Initial results demonstrated that patients

			were able to recognize and correct the distortions as accurately as a healthy control.
Schmitz et al. 2014	Upper extremities; Stroke	A wireless wearable system that provides auditory feedback in real-time about arm-movements. The system is equipped with eleven inertial sensor units that can be attached to the back of the fingers, back of the hands, upper and lower arms, shoulders and the trunk, transmitting orientation and acceleration data to a portable computer.	The pilot study presented a sonification system for the upper extremities. The sonification was designed to address gross motor skills and provided four-dimensional information about arm positions and trajectories. Preliminary results showed significant improvements in gross motor skills of the paretic upper limb, warranting further testing with larger controlled trials.
Schmitz et al. 2018	Upper extremities; Stroke		The article presented the protocol for a randomized controlled trial that expands previous preliminary results (Schmitz et al. 2014). Patients will undertake a total of 12 therapy sessions comprising gross motor arm movement training, including reaching, grasping, bimanual activities, and velocity. Patients allocated to the experimental group will receive real-time auditory feedback concomitant with the movement, so that arm elevation increases the sound frequency and wrist velocity changes sound amplitude, for instance. On the other hand, for the control group, arm movements will produce the sound of ocean waves. The hypothesis tested in this study is that patients in the experimental group will significantly improve in measures of gross motor function, exceeding changes found in the control group. Clinical results are yet to be reported.
Scholz et al. 2015	Upper extremities; Stroke	Musical sonification system. Patients' arm movements were sonified in real-time using two inertial sensors placed at the wrist and upper-arm of the affected side.	This pilot study tested a musical sonification therapy designed to retrain gross-motor functions. The 3D-movement data were transformed into music (vertical direction into discrete pitches, horizontal into sound timbre and sagittal into the sound volume) informing the patients about their arm position. Preliminary results indicated that

			patients in the music group showed improved motor function after 9 sessions encouraging further investigations of the feasibility and effectiveness of this musical sonification system (see Scholz et al., 2016).
Scholz et al. 2016	Upper extremities; Stroke	Musical sonification system	The study is based on Scholz et al. 2015 but expanding the study sample. The results showed that a sub-group of participants in the music group improved significantly in measures of pain, and there was a trend towards changes in motor junction after ten training sessions (20 min each) with musical sonification.
Tissberger and Wersenyi, 2011	Balance and lower extremities movement	Two sonification prototypes were investigated: a balance coordination system and a robotic system to assist lower extremities movement.	This publication overviewed two sonification approaches. The first addressed incorrect body posture in a standing position and the unbalanced distribution of the body mass on the legs by mapping front-back asymmetries via pitch changes up/down and unbalanced position by increasing feedback loudness.<br><br>The second system is a walking robot that helps patients paralyzed or restricted below the waistline to re-learn walking for diagnostic purposes and therapy. The mechanic system moves the legs of the patient and monitors the motion activity of the muscles. During sonification, a reference sound of the robot movement is presented and the patient's task was to reproduce the sound with his own body movement. Sonification time frames were discussed but no empirical data was reported.
Torres et al. 2013	Gait	Sonification system composed by a hardware part consisting of a foot glove-sole enhanced with sensors and a wearable IMU-System for motion tracking from the lower limb, and software composed of a motion analysis module, a sonification	The study presented a system prototype for sonification of gait patterns and a tool that facilitates the process of sonic interaction design for walking apparatuses. Preliminary testing with healthy individual showed potential for the implementation of motion-sound-coupling techniques, requiring further testing with patient populations.

		module, and an experiment aid module.	
Vogt et al. 2010	Upper extremities	PhysioSonic - a training system equipped with a VICON-motion-tracking system to measure 3D movements of the shoulder joint and sonify simple and intuitive attributes of the body movement (e.g., absolute height, velocity).	This pilot study tested a training system – PhysioSonic – that provides auditory feedback for orthopedic patients that have difficulties in raising their arms laterally. Initial testing suggested improvement of movement execution (4.5-degree improvements in arm-abduction) and an optimized (reduced) evasive movement of the contralateral upper-limb when using the PhysioSonic system.
Wallis et al. 2007	Upper extremities; Stroke	Biofeedback system that incorporates musical feedback as a means to maintain patient interest and provide movement information.	The study provided preliminary results on the effectiveness of a biofeedback system designed to assist in upper extremities rehabilitation. The system consists of marker-based motion capture and real-time analysis of movement features providing auditory feedback designed to help patients improve motions such as reach, openness, and flow. For instance, orchestra sounds were linked to elbow openness, whereas audio alerts were used to ensure minimal movement compensation thereby improving joint usage. Initial results with healthy volunteers and stroke patients showed the feasibility of this sonification system.
Young et al. 2014	Gait; Parkinson's Disease		The study investigated whether ecological sounds (e.g., footsteps on gravel) could be used in sonification systems to convey spatial and temporal parameters of gait. For that, patients were presented with recordings of sounds (condition 1: recordings of natural sounds, condition 2: synthesized sounds) and were asked to re-enact both the spatial and temporal parameters of the sounds. The results suggested that PD patients were able to accurately perceive and re-enact the spatial and temporal characteristics of gait with ecologically-valid sounds (condition 1), but synthetic sounds were insufficient to evoke similar changes in behavior.
